# Levodopa and Plant‐Derived Bioactive Compounds in Parkinson's Disease: Mechanisms, Efficacy, and Future Perspectives

**DOI:** 10.1111/cns.70540

**Published:** 2025-08-13

**Authors:** Emre Aktaş, Haşmet Ayhan Hanağası, Nehir Özdemir Özgentürk

**Affiliations:** ^1^ Faculty of Art and Science, Molecular Biology and Genetics Yıldız Technical University Istanbul Turkey; ^2^ Behavioral Neurology and Movement Disorders Unit, Department of Neurology, Istanbul Faculty of Medicine Istanbul University Istanbul Turkey

**Keywords:** levodopa and motor fluctuations, oxidative stress and neuroinflammation in PD, Parkinson's disease therapy, phytochemicals as adjuncts in PD treatment, plant‐derived bioactive compounds in Parkinson's disease, plant‐derived neuroprotective compounds

## Abstract

**Background:**

Parkinson's disease (PD) is a progressive neurodegenerative disorder characterized by the degeneration of dopaminergic neurons in the substantia nigra, resulting in dopamine deficiency and motor dysfunction. While levodopa (L‐DOPA) remains the gold standard for symptomatic treatment, its long‐term administration is associated with complications such as motor fluctuations, dyskinesia, and oxidative stress. Given these limitations, interest has grown in plant‐derived bioactive compounds for their potential neuroprotective and disease‐modifying effects.

**Methods:**

A systematic literature review was conducted across PubMed, Scopus, Google Scholar, and Web of Science, focusing on peer‐reviewed studies published between 2023 and March 2025. The inclusion criteria targeted in vitro and in vivo preclinical studies as well as clinical trials that directly compared levodopa with plant‐derived compounds in the context of PD. Key search terms included “Parkinson's disease,” “levodopa,” “phytochemicals,” and “plant‐based neuroprotection”.

**Results:**

Recent studies have highlighted several classes of plant‐based compounds—including polyphenols (resveratrol, curcumin, EGCG), flavonoids (quercetin, apigenin, naringenin), alkaloids (berberine, caffeine, L‐DOPA derived from *Mucuna pruriens*), and terpenoids (ginkgolide B, celastrol)—as potential neuroprotective agents. These compounds exert multiple actions, such as reducing oxidative stress, blocking neuroinflammation, preventing α‐synuclein aggregation, and protecting mitochondria. Although levodopa effectively addresses motor symptoms, these phytochemicals may complement conventional therapy by targeting underlying disease processes.

**Conclusions:**

Although levodopa is indispensable for the symptomatic management of PD, emerging evidence supports the integration of plant‐derived bioactive compounds as adjunct therapies with disease‐modifying potential. Future research should prioritize improving bioavailability, developing standardized formulations, and conducting long‐term clinical trials to evaluate the translational applicability of these natural agents in Parkinson's disease therapy.

## Graphical abstract

Plant‐derived compounds such as polyphenols, flavonoids, alkaloids, and terpenoids exhibit neuroprotective, antioxidant, and anti‐inflammatory activities in Parkinson's disease models. Unlike levodopa, they provide disease‐modifying benefits by targeting α‐synuclein aggregation, mitochondrial dysfunction, and neuroinflammation.

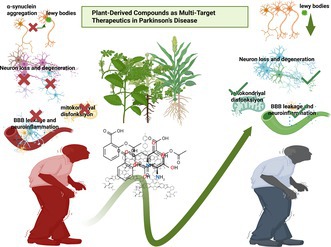

## 1 Introduction

Parkinson's disease is the second most common neurodegenerative disorder, affecting millions worldwide [1]. The main pathological hallmark of PD is the degeneration of dopaminergic neurons in the substantia nigra, resulting in dopamine deficiency and subsequent motor symptoms like bradykinesia, rigidity, and tremors [2]. Many drugs are available to treat PD, such as levodopa (L‐DOPA), catechol‐O‐methyltransferase (COMT) inhibitors, monoamine oxidase‐B (MAO‐B) inhibitors, and dopamine agonists [3]. In PD, COMT inhibitors, MAO‐B inhibitors, and dopamine agonists play crucial roles in managing motor symptoms by modulating dopamine levels and function within the basal ganglia [4, 5]. COMT is an enzyme responsible for the breakdown of levodopa in the peripheral and central nervous systems. COMT inhibitors (e.g., entacapone, tolcapone, opicapone) prolong levodopa's half‐life by preventing its peripheral degradation, increasing its availability in the brain, and enhancing the duration of dopamine action [6]. They are primarily used as adjunct therapy to levodopa to reduce motor fluctuations, particularly the “wearing‐off” phenomenon [3, 6]. MAO‐B is an enzyme that degrades dopamine in the brain. MAO‐B inhibitors (e.g., selegiline, rasagiline, safinamide) prevent dopamine breakdown, thereby prolonging dopaminergic activity and reducing oxidative stress, which is implicated in PD progression [7]. These drugs are often used in early‐stage PD as monotherapy or as an adjunct to levodopa in later stages to enhance its effectiveness [7, 8]. Dopamine agonists are drugs that directly stimulate dopamine receptors (D1‐ and D2‐like receptors) in the striatum, mimicking endogenous dopamine activity [9]. Examples include pramipexole, ropinirole, and rotigotine [10]. Dopamine agonists are often used in early PD or as an adjunct to levodopa to delay motor complications, particularly in younger patients [3, 9, 10]. Overall, COMT inhibitors and MAO‐B inhibitors are used in combination with levodopa to prolong its effects and reduce fluctuations, and dopamine agonists can delay the need for levodopa in early disease stages and reduce dyskinesia risk—though they may cause impulse control disorders and hallucinations as side effects [6, 9, 10]. As evident from the above, these pharmacological agents primarily function as adjunct therapies to levodopa, which remains the cornerstone of Parkinson's disease treatment [11]. However, long‐term use of synthetic L‐DOPA is linked to complications such as motor fluctuations, dyskinesias [11]. Yet, even with these medications, current therapies simply compensate for dopamine loss and do not completely suppress PD symptoms [12]. Therefore, scientists have increasingly turned their attention to natural compounds, which may offer more promising anti‐parkinsonian potential [11, 13]. Because recent studies highlight that plant‐derived bioactive compounds may not only alleviate symptoms in Parkinson's disease but also offer disease‐modifying potential through multi‐target mechanisms, such as oxidative stress inhibition, anti‐inflammatory activity, and mitochondrial protection [11, 13, 14, 15, 16]. Unlike levodopa, which primarily restores dopaminergic tone, these natural compounds may act on non‐dopaminergic pathways that are also implicated in disease progression. When used in combination with levodopa, certain phytochemicals(such as curcumin, resveratrol, and quercetin) have demonstrated synergistic effects, potentially enhancing motor function, reducing levodopa‐induced toxicity, and prolonging its therapeutic efficacy. These findings suggest that natural compounds may represent a promising adjunctive strategy to address the limitations of current mono‐therapies and support a more holistic, long‐term approach to PD management [13, 14, 15, 16, 17].

Many studies have shown that various medicinal plants and natural bioactive compounds possess antioxidant and anti‐inflammatory properties, making them promising candidates for antiparkinsonian therapy with minimal side effects [14, 15, 16, 17]. In recent years, plant‐derived bioactive compounds have gained attention as potential alternatives or adjunct therapies to levodopa due to their neuroprotective, antioxidant, and anti‐inflammatory properties [14, 15]. Until now, numerous bioactive molecules with recognized neuroprotective mechanisms—such as polyphenols, terpenes, and alkaloids—have been extracted from plants and identified for their therapeutic potential in PD. However, there remains a lack of comprehensive and up‐to‐date reviews consolidating emerging trends in potent natural bioactive compounds for PD management. This review aims to address that gap by focusing only on recent (2023 to March 10, 2025) peer‐reviewed literature, ensuring an accurate and current analysis that reflects the rapidly evolving landscape of PD research. Staying current is crucial, as the global prevalence of Parkinson's disease is projected to rise from about 6.2 million cases in 2015 to roughly 25.2 million by 2050, representing a 112% increase from estimates in 2021, according to the latest projections from a study [18]. Furthermore, with an increasing aging population, the impact of PD will become even more pronounced [19]. In summary, plant‐derived bioactive compounds have gained momentum as potential alternatives or adjuncts to levodopa, owing to their multipronged neuroprotective effects. This review compares levodopa with prominent categories of such compounds—polyphenols, flavonoids, alkaloids, and terpenoids—highlighting their mechanisms of action, efficacy, pharmacokinetics, and safety profiles, with an emphasis on studies published between 2023 and 2025. The goal is to evaluate how these natural compounds might complement or enhance current PD therapy and to identify future directions for integrating natural therapeutics into PD management.

## 2 Methodology

**FIGURE 1:**
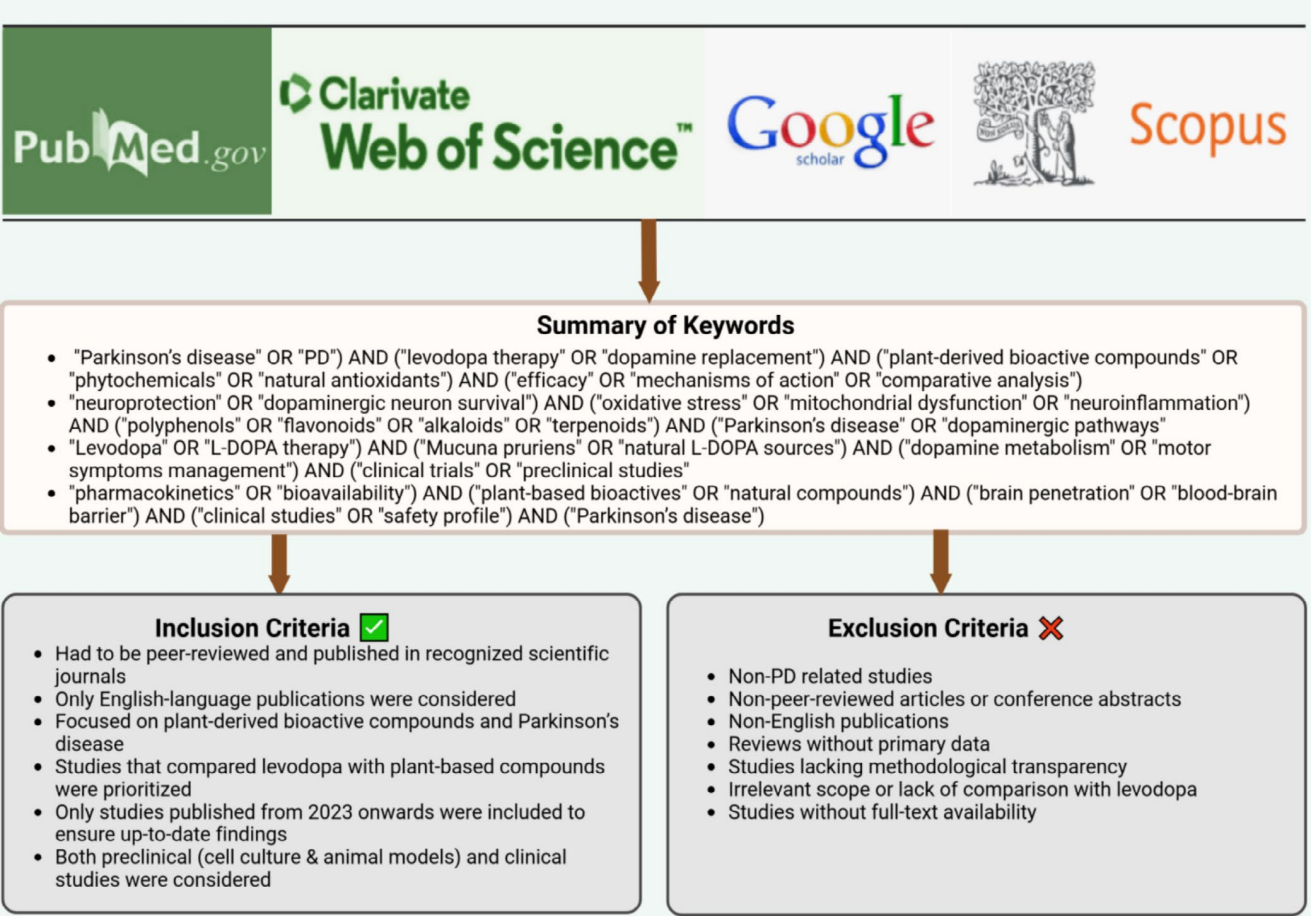
Shows the systematic approach employed in this review to identify relevant studies on plant‐based bioactive compounds for the treatment of Parkinson's disease.

A systematic literature search was conducted to explore the therapeutic potential of plant‐based bioactive compounds in Parkinson's disease. The search was performed across PubMed, Scopus, Google Scholar, and Web of Science, utilizing a combination of relevant keywords, such as “Parkinson's disease,” “plant‐based bioactive compounds in PD,” “phytochemicals in Parkinson's disease,” “dopaminergic neuroprotection,” “comparison of levodopa and plant‐derived compounds,” and “recent comparisons of levodopa with plant‐derived therapeutics.” The inclusion criteria required that selected studies be peer‐reviewed and published in English in reputable scientific journals. Studies had to explicitly focus on the relationship between plant‐derived bioactive compounds and PD, directly comparing levodopa and plant‐based compounds in in vitro or in vivo models. To ensure relevance and scientific rigor, only studies published from 2023 onwards were considered. Both preclinical models (cell culture and animal studies) and clinical trials were included to provide a comprehensive evaluation of current evidence. The exclusion criteria eliminated studies that were not directly related to PD pathophysiology or therapeutic applications. Additionally, research lacking methodological robustness, such as non‐peer‐reviewed articles, conference abstracts, or non‐English publications, was excluded. Studies that did not provide full‐text availability or failed to offer a clear comparison between levodopa and plant‐based compounds were also removed from consideration. This systematic approach (Figure 1) aimed to synthesize high‐quality evidence while minimizing bias. This study provides a comparative perspective on the potential role of plant‐derived bioactive compounds alongside conventional levodopa treatment in PD management by critically assessing their neuroprotective and therapeutic roles.

## 3 Neuropathological Features of Parkinson's Disease

**FIGURE 2:**
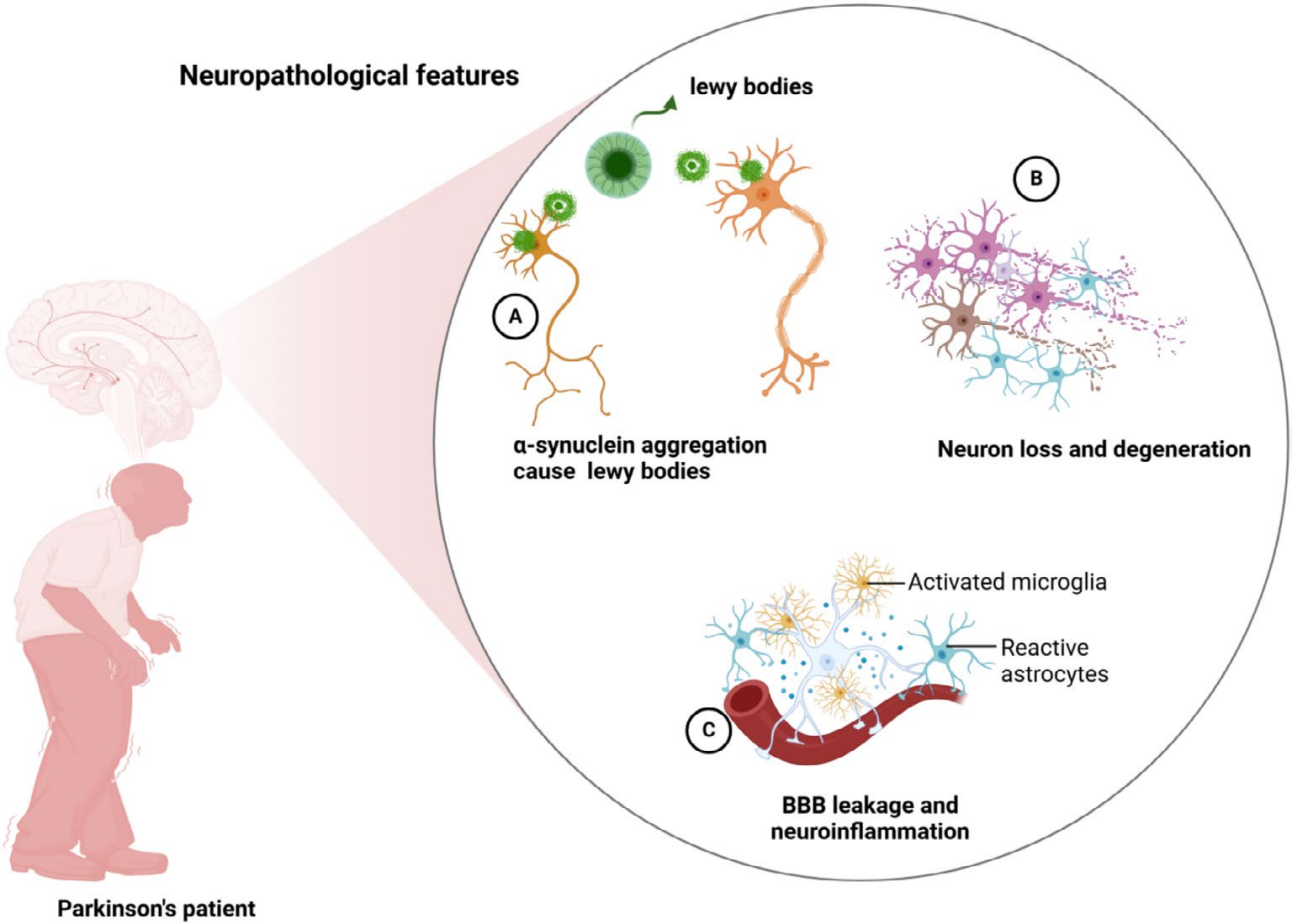
Overview of neuropathological changes in Parkinson's disease (PD). The figure highlights (A) intracellular α‐synuclein aggregation and Lewy body formation, (B) dopaminergic neuron loss, especially in the substantia nigra, and (C) blood–brain barrier disruption with associated neuroinflammation.

Parkinson's disease is characterized by distinct neuropathological hallmarks, primarily involving intracellular Lewy bodies and dopaminergic neuron degeneration. Lewy bodies, primarily composed of misfolded α‐synuclein, aggregate within neurons, disrupting cellular proteostasis and damaging mitochondria, thus promoting neuronal toxicity (Figure 2) [5]. The progressive degeneration of dopaminergic neurons in the substantia nigra pars compacta leads directly to dopamine deficiency, underpinning the hallmark motor symptoms such as bradykinesia, resting tremor, and rigidity, as well as contributing to non‐motor symptoms including autonomic, mood, and cognitive disorders [2]. Moreover, neuroinflammation, characterized by activated microglia and reactive astrocytes, significantly contributes to PD pathology by releasing pro‐inflammatory cytokines and reactive oxygen species, thereby exacerbating neuronal injury (Figure 2) [20, 21]. Additionally, blood–brain barrier (BBB) dysfunction, observed through increased permeability and leakage of inflammatory mediators into the brain, further enhances neuroinflammation and neuronal vulnerability [21]. Lastly, oxidative stress and mitochondrial dysfunction, prominently arising from dopamine auto‐oxidation and impaired mitophagy pathways (e.g., mutations in PINK1, PRKN), are central to neuronal degeneration in PD, emphasizing their importance as therapeutic targets [22, 23].

## 4 Etiological Factors in Parkinson's Disease

**FIGURE 3:**
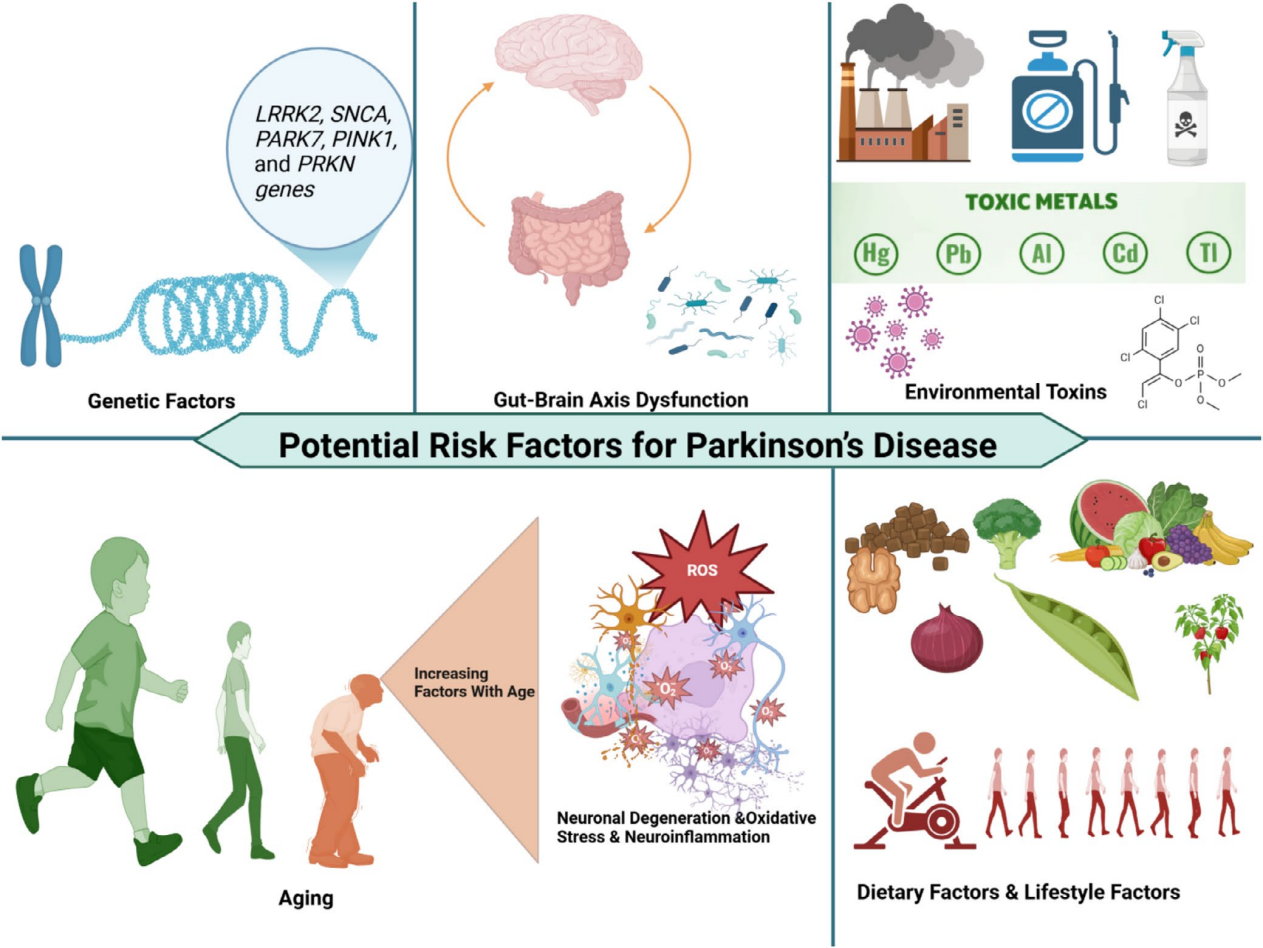
Key risk factors associated with Parkinson's disease (PD). The diagram summarizes major contributors to PD pathogenesis, including genetic mutations (e.g., LRRK2, SNCA), environmental toxins (e.g., heavy metals), aging‐related changes, dietary and lifestyle factors, oxidative stress, and gut–brain axis dysfunction.

Parkinson's disease is a multifactorial neurodegenerative disorder arising from a complex interplay of genetic predispositions, environmental influences, aging, and dietary factors (Figure 3). Familial Parkinson's disease cases, which account for approximately 5%–10% of all cases, are associated with mutations in genes such as SNCA, LRRK2, PINK1, and PRKN [24]. These genes regulate essential cellular processes, including mitochondrial function, autophagy, lysosomal activity, proteasomal degradation, cellular stress responses, inflammation, and synaptic homeostasis, all of which contribute to the pathogenesis of the disease [24]. Sporadic PD cases commonly involve oxidative stress and chronic neuroinflammation, driven by reactive oxygen species (ROS), microglial activation, and mitochondrial impairment, which collectively exacerbate neuronal degeneration [20, 25]. Emerging evidence also implicates gut‐brain axis dysfunction and microbiome dysbiosis in PD, where peripheral α‐synuclein pathology and intestinal inflammation may propagate neurodegeneration via vagal pathways [26]. Environmental exposures, including pesticides (rotenone, paraquat), heavy metals (manganese, mercury, lead), and air pollutants, significantly increase PD risk through oxidative mechanisms and inflammatory pathways [27]. Aging remains the most prominent risk factor, amplifying neuronal susceptibility due to diminished proteostasis, mitochondrial function, and blood–brain barrier integrity [28]. Dietary patterns further modulate PD risk; antioxidant‐rich diets (Mediterranean diet, flavonoids) provide neuroprotection, whereas diets high in saturated fats, cholesterol, pesticides, or heavy metals elevate vulnerability [29, 30, 31]. Collectively, these interconnected factors underline the complexity of PD etiology and provide avenues for therapeutic intervention (Figure 3).

## 5 Plant‐Based Bioactives

**FIGURE 4:**
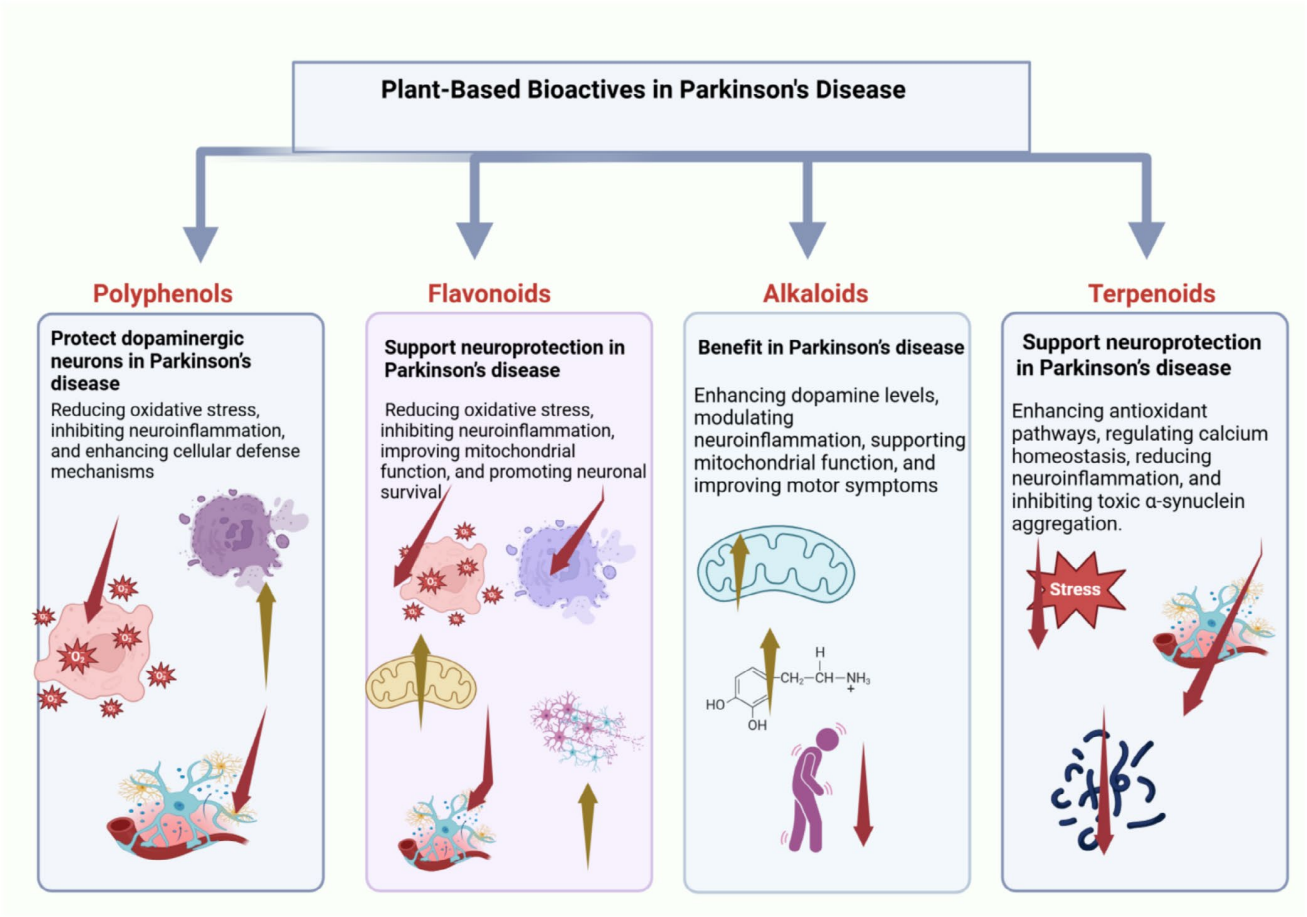
Mechanisms of action of plant‐based bioactive compounds in Parkinson's disease. Overview of the neuroprotective effects exerted by polyphenols, flavonoids, alkaloids, and terpenoids in PD pathogenesis, highlighting their therapeutic potential through multiple cellular pathways. An upward arrow (↑) is used to indicate an increase, enhancement, stimulation, or facilitation of a process, function, or biological activity. Conversely, a downward arrow (↓) signifies a decrease, inhibition, suppression, or attenuation of a given process.

Within the rich diversity of plant‐derived chemicals, we focus on four major classes with reported anti‐parkinsonian potential: polyphenols, flavonoids, alkaloids, and terpenoids. Below, we summarize key examples from each class and their known neuroprotective effects relevant to PD (Figure 4).

### 5.1 Polyphenols

This class includes compounds like resveratrol, curcumin, and epigallocatechin‐3‐gallate (EGCG). Resveratrol (a stilbene from grapes/red wine) and curcumin (a diarylheptanoid from the spice turmeric) are known for strong antioxidant and anti‐inflammatory properties [31, 32]. Epidemiological observations suggest that diets rich in polyphenols (e.g., berries, tea, red wine) are associated with reduced PD risk. In PD models, resveratrol and related polyphenols protect neurons via antioxidant effects (scavenging reactive oxygen species) and by activating cellular defense enzymes [14, 33]. Studies have shown that resveratrol can activate the SIRT1 pathway, improve mitochondrial function, and reduce α‐synuclein aggregation [27, 34]. Curcumin (from *Curcuma longa* ) inhibits oxidative stress, enhances levels of brain‐derived neurotrophic factor (BDNF), and mitigates inflammation in PD models [35, 36, 37]. Likewise, a green tea polyphenol, EGCG, protects dopaminergic neurons by reducing oxidative stress and inhibiting neuroinflammation [20].

### 5.2 Flavonoids

Flavonoids are a subclass of polyphenols abundant in fruits and vegetables (e.g., quercetin, apigenin, catechin, naringenin, fisetin). Higher flavonoid intake has been epidemiologically linked to lower PD incidence and slower progression [29, 38]. For example, small clinical trials using flavonoid‐rich foods (like cocoa or licorice) showed modest motor improvements in PD patients [38]. Quercetin (found in apples, onions, and tea) exhibits neuroprotective effects in toxin‐induced PD models by reducing oxidative stress, particularly in the cerebellum [15, 39] Clinical interest in flavonoids is growing. For instance, supplementation with flavonoid‐rich foods such as cocoa or licorice has shown potential benefits on motor function in preliminary studies [38]. Apigenin (from parsley and chamomile) has notable anti‐inflammatory properties and promotes neuronal survival, while naringenin (from citrus fruits) improves mitochondrial function and can increase dopamine levels in models [34, 40]. Fisetin, a bioactive flavonoid primarily found in strawberries, apples, and persimmons, demonstrates significant neuroprotective properties due to its powerful antioxidant and anti‐inflammatory effects [28]. Recent studies have emphasized its capability to modulate various molecular pathways associated with neurodegenerative diseases, including Parkinson's disease. Fisetin provides neuroprotective benefits by scavenging reactive oxygen species, inhibiting pro‐inflammatory cytokines, and promoting neuronal survival pathways [41]. Moreover, fisetin has been shown to alleviate oxidative stress caused by transition metals, a major contributor to neurodegeneration in PD, by chelating metal ions and diminishing metal‐catalyzed lipid peroxidation and protein aggregation [42].

### 5.3 Alkaloids

Plant alkaloids with potential benefits in PD include berberine, caffeine, and nicotine, among others. Berberine (from Berberis species) is an isoquinoline alkaloid with broad neuroprotective mechanisms (antioxidant, anti‐apoptotic, anti‐inflammatory) [43]. Oral berberine can elevate brain dopamine levels and improve motor function in PD model animals, partly via altering the gut microbiome [26, 43] It also modulates autophagy and mitochondrial function, reducing dopaminergic neuron loss in PD models [44]. Caffeine (a xanthine alkaloid found in coffee and tea) is epidemiologically linked to lower PD risk; it acts as an adenosine A_2_A receptor antagonist, which may indirectly support dopaminergic signaling [45]. *Mucuna pruriens* (velvet bean) seeds deserve special mention: they naturally contain L‐DOPA (the same molecule as synthetic levodopa) and other alkaloids [46]. Mucuna has long been used in traditional Ayurvedic medicine for parkinsonian symptoms and is being re‐investigated in modern research [47].

### 5.4 Terpenoids

Terpenoids (isoprene‐based compounds) from various herbs have shown neuroprotective effects. Examples include carnosic acid (a diterpene from rosemary), ginkgolide B (a diterpene from *Ginkgo biloba* ), and celastrol (a triterpene from Tripterygium vine). Carnosic acid enhances endogenous antioxidant pathways in neurons via Nrf2 activation and increases neurotrophic factors like BDNF [48]. Ginkgolide B can protect dopaminergic cells by reducing intracellular calcium overload and caspase‐3 activity, and by restoring levels of calbindin (a calcium‐binding protein), thereby aiding neuronal survival [49]. Ginkgolide B also helps shield neurons from oxidative damage and reduces neuroinflammation [50]. Celastrol suppresses neuroinflammation by inhibiting NF‐κB signaling, thereby reducing pro‐inflammatory cytokines and even toxic α‐synuclein species [51]. Post‐2022 studies have particularly explored novel combinations and less‐studied compounds. For example, one 2023 study proposed a curcumin–levodopa nanoparticle formulation to harness curcumin's neuroprotective effect alongside dopamine replacement [52]. Collectively, these natural compounds represent diverse chemical classes with multi‐faceted bioactivities relevant to PD.

## 6 Mechanisms of Action: Levodopa and Natural Compounds

### 6.1 Dopaminergic Pathways

**FIGURE 5:**
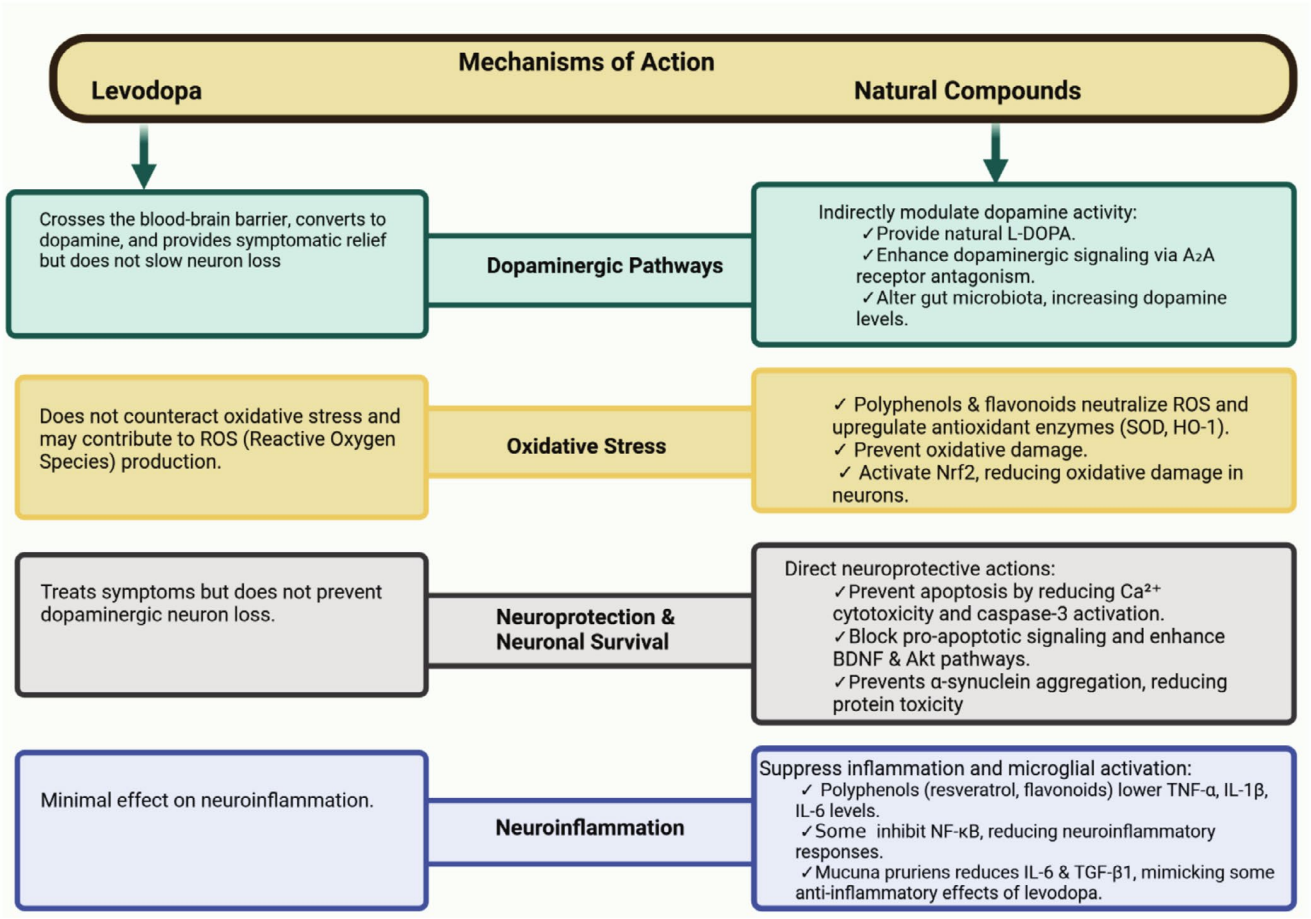
Comparative mechanisms of action: Levodopa versus natural bioactive compounds in Parkinson's disease.

Levodopa's primary mechanism is straightforward—as the metabolic precursor to dopamine, it crosses the blood–brain barrier and is converted to dopamine, thereby replenishing the neurotransmitter that is deficient in PD [21]. This leads to significant relief of motor symptoms. However, levodopa mainly addresses dopamine replacement and does not directly intervene in the degenerative process; it is well documented that conventional dopaminergic drugs (levodopa and others) provide symptomatic benefit but do not halt or slow the ongoing loss of nigral neurons [4]. In contrast, most plant‐based compounds do not directly increase brain dopamine levels (with the notable exception of *Mucuna pruriens* delivering natural L‐DOPA) [53]. Instead, natural bioactives tend to modulate dopaminergic pathways indirectly or preserve dopaminergic neurons [37, 53, 54, 55]. For example, caffeine's antagonism of adenosine A_2_A receptors in the striatum can enhance dopaminergic signaling and has been associated with lower PD incidence [45]. Another example is berberine—while not a dopamine precursor, it was shown to increase striatal dopamine in PD‐model mice by altering gut microbiota composition (increasing certain gut bacteria that produce dopamine or support host dopamine levels) [26, 43, 56]. Thus, some natural compounds can influence dopamine availability or receptor activity in unconventional ways (Figure 5).

### 6.2 Oxidative Stress

Oxidative damage is a key contributor to PD pathology, and this is an area where natural compounds markedly differ from levodopa [57]. Levodopa itself does little to combat oxidative stress—in fact, the oxidative metabolism of dopamine (and of levodopa in peripheral tissues) can generate reactive oxygen species and toxic dopamine‐quinones [58]. There is evidence that the oxidation of L‐DOPA can produce free radicals that may exacerbate neuronal degeneration [59, 60, 61]. Natural antioxidants, on the other hand, directly counteract oxidative stress. Polyphenols and flavonoids are well‐known free radical scavengers and also boost endogenous antioxidant defenses [15, 34]. Resveratrol, quercetin, and apigenin have been reported to neutralize ROS and upregulate antioxidant enzymes like superoxide dismutase (SOD) and heme oxygenase‐1 (HO^−1^) in cellular PD models [39, 62]. Curcumin likewise activates the Nrf2 pathway, which increases expression of antioxidant genes, and it inhibits lipid peroxidation and nitric oxide production in toxin‐based PD models [22, 36]. In a rotenone‐induced PD rat study (2023), the alkaloid berberine significantly reduced markers of oxidative stress (nitric oxide and lipid peroxides) in the striatum while improving mitochondrial function—effects attributed to Nrf2 antioxidant pathway activation [43, 44]. In summary, unlike levodopa, many phytochemicals mitigate the oxidative damage underlying PD, which may help protect neurons rather than just temporarily relieve symptoms [14, 63, 64].

### 6.3 Neuroprotection and Neuronal Survival

Levodopa does not confer neuroprotective effects—it treats the symptoms resulting from dopaminergic cell loss but does not prevent that loss [65]. By contrast, plant‐derived compounds often exhibit direct neuroprotective mechanisms. For instance, terpenoids like ginkgolide B and celastrol can promote the survival of dopaminergic neurons by modulating cell‐death pathways (Figure 5) [66]. Ginkgolide B reduces Ca^2+^‐mediated cytotoxicity and caspase‐3 activation in neurons, which helps prevent apoptosis of dopaminergic cells [49]. Polyphenols can inhibit pro‐apoptotic signaling: resveratrol has been shown to lower active caspase‐3 levels and prevent neuronal death in pesticide‐induced PD cell models [14, 27]. Curcumin exerts multi‐targeted neuroprotective actions, ranging from blocking the aggregation of toxic α‐synuclein protein to activating pro‐survival signaling pathways (e.g., PI3K/Akt and BDNF pathways) [48, 67]. These actions can preserve neuronal integrity. There is even evidence that curcumin and its analogues prevent the formation of α‐synuclein fibrils and can destabilize existing aggregates [68], potentially slowing the underlying disease process. In summary, neuroprotective capacity is a major differentiator: natural compounds engage pathways (antioxidant responses, anti‐apoptosis, protein homeostasis, etc.) that maintain neuron health, whereas levodopa largely bypasses these disease‐modifying mechanisms (Figure 5). This fundamental difference suggests that natural bioactives could play a role in disease modification or serve as adjuncts to create a more neuroprotective neuronal milieu—something levodopa alone cannot achieve [14, 63].

### 6.4 Neuroinflammation

Chronic neuroinflammation (activation of microglia and release of cytokines) contributes to PD progression [69]. Levodopa is not known to significantly reduce neuroinflammation; it primarily works at the level of neurotransmission and symptom relief [70]. By contrast, many plant compounds exhibit anti‐inflammatory effects in the brain [71, 72]. Polyphenols like resveratrol and various flavonoids attenuate microglial activation and lower levels of pro‐inflammatory cytokines (such as TNF‐α, IL‐1β, IL‐6) in experimental models [15, 34]. In a mouse PD model, *Mucuna pruriens* seed extract (rich in natural L‐DOPA plus other constituents) was found to decrease levels of interleukin‐6 (IL‐6) and TGF‐β1 in the brain, similar to pure levodopa [47]. This suggests that both levodopa and the Mucuna extract mitigated certain inflammatory markers in that scenario. However, many other phytochemicals more directly target inflammation: celastrol, for example, powerfully inhibits NF‐κB, a master regulator of inflammation, thereby reducing downstream cytokines and slowing neurodegenerative inflammation [51]. Berberine likewise suppresses inflammatory responses; it inhibits microglial activation and has been shown to reduce serum levels of inflammatory cytokines in toxin‐induced parkinsonian rats [43, 44]. Overall, natural compounds tend to create a more neuroprotective environment by dampening neuroinflammation, whereas levodopa's effect on inflammation is minimal or secondary. In summary, levodopa's mechanism is singular and symptomatic—restoration of dopamine transmission. Natural compounds, in contrast, exert a broad spectrum of actions: anti‐oxidative stress, anti‐inflammatory effects, anti‐apoptotic and neurotrophic support, and modulation of protein aggregates [63]. These mechanisms complement the dopaminergic pathway and address upstream pathogenic processes. This fundamental difference suggests that natural bioactives could contribute to disease modification or serve as adjuncts to make the neuronal milieu more favorable—outcomes that levodopa alone cannot achieve [14, 63].

## 7 Efficacy in Preclinical and Clinical Studies

### 7.1 Preclinical (In Vitro and In Vivo) Evidence

**FIGURE 6:**
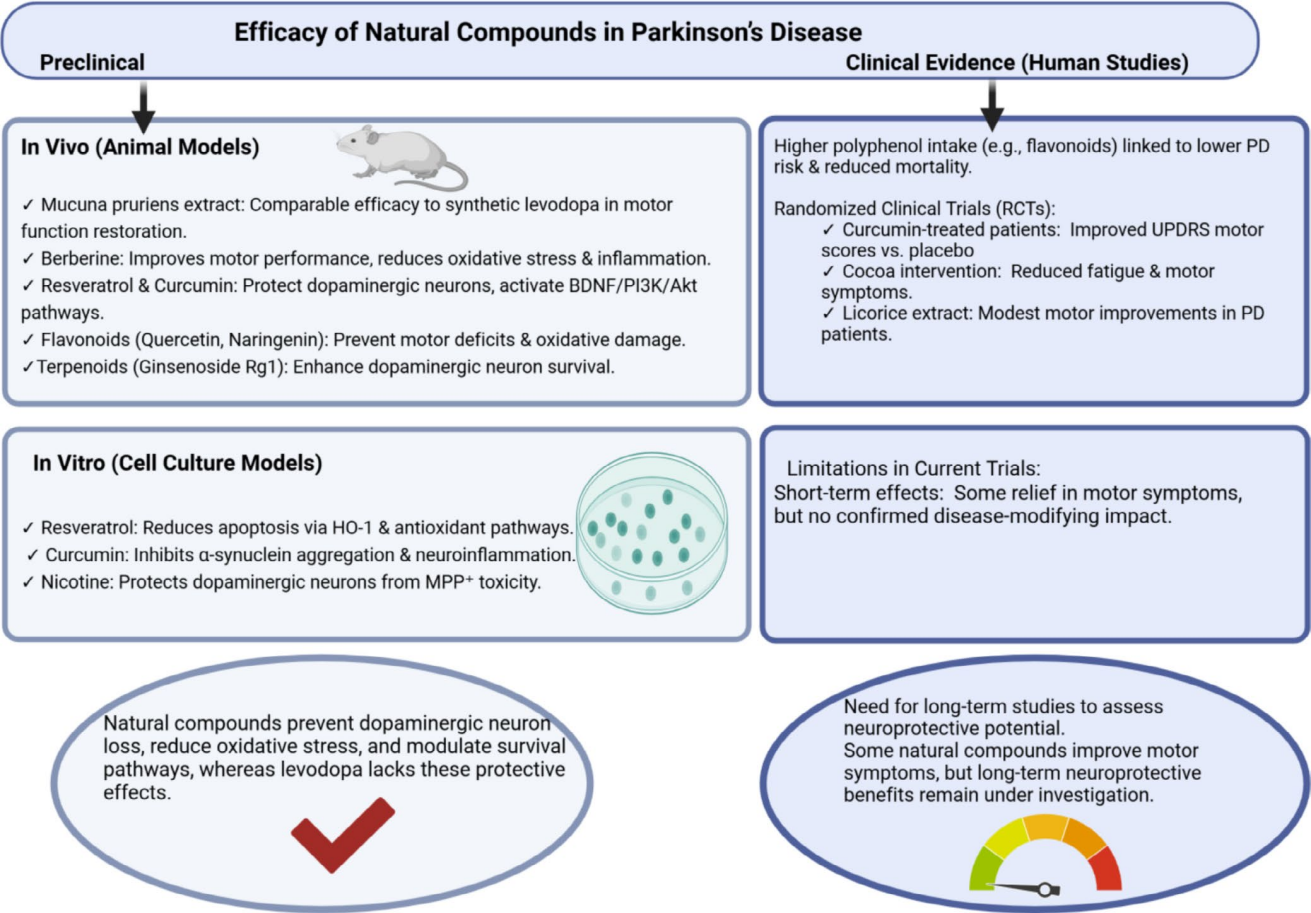
Efficacy of natural bioactive compounds in Parkinson's disease: evidence from preclinical and clinical studies.

A growing body of recent preclinical research demonstrates that plant‐based compounds can ameliorate PD‐like pathology in laboratory models [73]. In toxin‐induced rodent models of PD, many natural products improve motor function, preserve dopaminergic neurons, and reduce biochemical markers of neurodegeneration (Figure 6). For example, in vivo studies have compared natural extracts to levodopa head‐to‐head. A 2023 study compared *Mucuna pruriens* seed extract to pure levodopa in a mouse PD model (rotenone‐intoxicated mice). Strikingly, when dosed to provide equivalent L‐DOPA content (the Mucuna extract naturally contains ~5% L‐DOPA by weight), the plant extract improved motor deficits to a similar extent as synthetic levodopa, with no statistically significant difference between the two treatments [47]. Both treatments rescued motor coordination (e.g., on beam balance and olfactory function tests) and lowered inflammatory cytokines in the brain, indicating that Mucuna's natural concoction is as efficacious as levodopa in that model [74]. This suggests that the additional phytochemicals in Mucuna did not diminish the antiparkinsonian effect—if anything, some studies imply they might enhance it. For example, another report noted *Mucuna pruriens* extract had roughly double the antiparkinsonian potency of pure levodopa in a rat model (measured by rotational behavior in a lesioned rat), possibly due to synergistic compounds in the extract boosting L‐DOPA's action. Beyond Mucuna, numerous animal studies of individual compounds show positive outcomes. Berberine (alkaloid) in a 2023 rat study prevented rotenone‐induced motor impairment: berberine‐treated PD rats performed better on locomotor and coordination tasks (open field, rotarod, etc.) compared to untreated PD rats [44]. Concurrently, berberine protected neurons by reducing oxidative stress and inflammation in the striatum, as described earlier. Resveratrol (polyphenol) has been extensively tested in PD models; a 2025 systematic review concluded resveratrol consistently exhibits neuroprotective effects in both cell cultures and various PD animal models, improving motor outcomes and reducing dopaminergic neuron loss [14, 34, 67]. Similarly, curcumin has shown efficacy in multiple rodent models, improving motor behavior and biochemical indices; studies demonstrated that curcumin alleviated motor dysfunction in a PD mouse model via activating BDNF/PI3K/Akt signaling [67]. Moreover, combining curcumin with levodopa (in nanoparticle form) was recently proposed as a strategy to provide both symptomatic relief and disease modification. Flavonoids like quercetin and naringenin have also improved motor functions in toxin‐based models, attributed to their antioxidative and anti‐apoptotic effects. For example, quercetin protected against pesticide‐induced motor deficits in rats by lowering oxidative damage in the cerebellum [75, 76]. Terpenoids such as ginsenoside Rg1 (from ginseng) have been reported to improve dopaminergic neuron survival and motor behavior in MPTP‐lesioned mice through anti‐inflammatory and anti‐oxidative mechanisms [63]. Overall, preclinical studies strongly support the efficacy of these natural compounds in reducing PD‐like neuropathology. While levodopa remains the most potent agent for reversing motor deficits in animal models (due to its direct dopaminergic effect), these adjunct natural therapies show significant protective or restorative effects that levodopa alone does not provide (Figure 6).

In vitro (cellular) studies also reinforce these findings. Cell culture models of PD (using neurotoxin‐treated neurons or genetic models) have been used to dissect mechanisms. Many natural compounds protect cultured dopaminergic neurons from toxins. For instance, in neuronal cells exposed to PD‐related stressors (like rotenone or α‐synuclein aggregates), polyphenols prevent cell death: resveratrol reduces apoptosis of PC12 dopaminergic cells by upregulating HO^−1^ and other antioxidant defenses [39, 62]. Curcumin inhibits inflammatory damage in microglial‐neuron co‐cultures and prevents α‐synuclein aggregation in vitro [36, 68]. Alkaloids such as nicotine can protect dopaminergic cells from MPP^+^ toxicity via activation of nicotinic receptors and downstream survival pathways—aligning with epidemiological hints that nicotine (from tobacco) may have neuroprotective effects in PD. These in vitro findings reinforce that natural molecules can directly safeguard neuronal cells, something not observed with levodopa in cell culture (levodopa in vitro can actually be toxic to neurons at high concentrations due to oxidative byproducts) [60, 61].

### 7.2 Clinical (Human) Studies

Clinical evidence for plant‐based compounds in PD is still emerging, but a few recent trials and observational studies offer insights.

### 7.3 Symptomatic Effects in Patients

A 2024 systematic review compiled results from the handful of randomized controlled trials (RCTs) and cohort studies available on polyphenol‐rich interventions in PD [38]. It found that higher dietary flavonoid and anthocyanin intake correlates with a lower risk of developing PD and even reduced mortality in PD patients, based on long‐term cohort data [38, 77]. On the interventional side, small RCTs reported that supplementation with licorice extract, curcumin, or cocoa (all rich in polyphenols) led to modest improvements in motor function in PD patients over the study period. For example, in one trial, curcumin‐treated patients showed better UPDRS motor scores compared to placebo (though no change in disease progression markers), and a cocoa intervention (high in flavanols) showed positive trends in reducing fatigue and motor symptoms [78]. Importantly, these short‐term trials did not find significant improvements in non‐motor symptoms or overall disease progression in the timeframes studied [38], indicating that while motor symptoms can be somewhat alleviated, more research is needed on long‐term neuroprotective outcomes.

### 7.4 Levodopa Versus Natural L‐DOPA Sources

There is growing clinical interest in *Mucuna pruriens* as an alternative levodopa source [53, 79]. Traditional Ayurvedic practice has used Mucuna seed powder in PD for centuries, and modern clinical studies (albeit few) have begun testing it. An earlier clinical study demonstrated that Mucuna seed powder had a faster onset of action than standard levodopa in PD patients and provided similar motor benefit without adverse effects in a single‐dose trial [80]. Recent reviews suggest that *Mucuna pruriens* could be used to help manage PD, potentially delaying the need for synthetic levodopa or smoothing out motor response fluctuations [2, 11, 53, 79, 80]. However, robust, large‐scale clinical trials are still lacking in this area (Figure 6).

### 7.5 Adjunct Therapy Trials

Some clinical trials are examining natural compounds as add‐ons to standard PD medications. For instance, trials of green tea polyphenols (EGCG) as an adjunct to the MAO‐B inhibitor rasagiline have been conducted to see if the combination can slow PD progression (results have been inconclusive) [30, 81, 82]. A study of nicotinamide riboside (a vitamin B_3_ derivative that elevates NAD^+^ levels and might support mitochondrial health) in PD is underway, reflecting a broader interest in metabolic nutrients for neuroprotection [83, 84]. While nicotinamide riboside is not a “plant extract” perse, this example highlights the broader category of bioactive nutrients being tested for disease‐modifying effects [16, 85]. In summary, levodopa remains unparalleled for immediate motor symptom relief in humans, which is why it is the gold‐standard therapy. No natural compound alone has yet shown equivalent symptomatic potency in established PD. Nonetheless, evidence from recent preclinical studies and early clinical trials indicates that natural bioactive compounds can be effective in complementary ways—modestly improving motor function, protecting neurons, and potentially slowing aspects of disease pathology (Figure 6). These benefits, observed in models and small patient trials, support the rationale for combining such compounds with levodopa therapy in the future. The efficacy data, while promising, also underscore that natural compounds are not a stand‐alone replacement for levodopa at present, but rather candidates for integrative approaches to PD management (Figure 6).

## 8 Side Effects and Safety Profile

One primary motivation for exploring natural alternatives in Parkinson's disease therapy is the adverse effect profile linked to chronic levodopa use. Long‐term use of levodopa frequently leads to debilitating motor complications, such as motor fluctuations (wearing‐off phenomena) and levodopa‐induced dyskinesias, attributed to pulsatile dopamine receptor stimulation and adaptive neural circuit changes [61, 86, 87]. Additional peripheral side effects such as nausea, vomiting, orthostatic hypotension, sleep disturbances, and neuropsychiatric symptoms further diminish patients' quality of life, particularly at higher doses or among elderly individuals [88, 89, 90]. In contrast, based on experimental findings, natural bioactive compounds typically demonstrate a favorable safety profile. Many plant‐derived supplements, including polyphenols and flavonoids, do not produce the common dopaminergic side effects, as demonstrated by clinical trials on curcumin and cocoa, which showed no worsening of motor fluctuations or dyskinesia [38]. Additionally, natural L‐DOPA sources like *Mucuna pruriens* , while carrying some dopamine‐related risks, exhibit a lower incidence of dyskinesia compared to synthetic levodopa, likely due to a more gradual dopamine release or the presence of additional modulatory compounds [2]. Overall, natural compounds show minimal systemic or organ toxicity at therapeutic doses, although mild side effects may occur—curcumin, for instance, has resulted in only minor gastrointestinal upset even at high doses [22, 30, 57]. Substances such as caffeine and nicotine present their own specific risks (e.g., insomnia or addiction), emphasizing that natural does not mean harmless; however, unlike levodopa, these natural compounds do not induce cumulative neurotoxicity, and many are neuroprotective against oxidative stress [60, 91, 92]. Clinical tolerability for natural supplements remains generally high, with systematic reviews reporting no serious adverse events, although potential drug interactions—such as polyphenols affecting levodopa metabolism—must be monitored under medical supervision [4, 38]. Thus, while comprehensive long‐term human data are needed, current evidence supports the safer long‐term use of natural bioactive compounds, either as adjunctive therapy to reduce levodopa dosage and associated side effects or possibly as preventive measures [93].

## 9 Pharmacokinetics and Bioavailability Between Levodopa and Plant‐Derived Compounds

**FIGURE 7:**
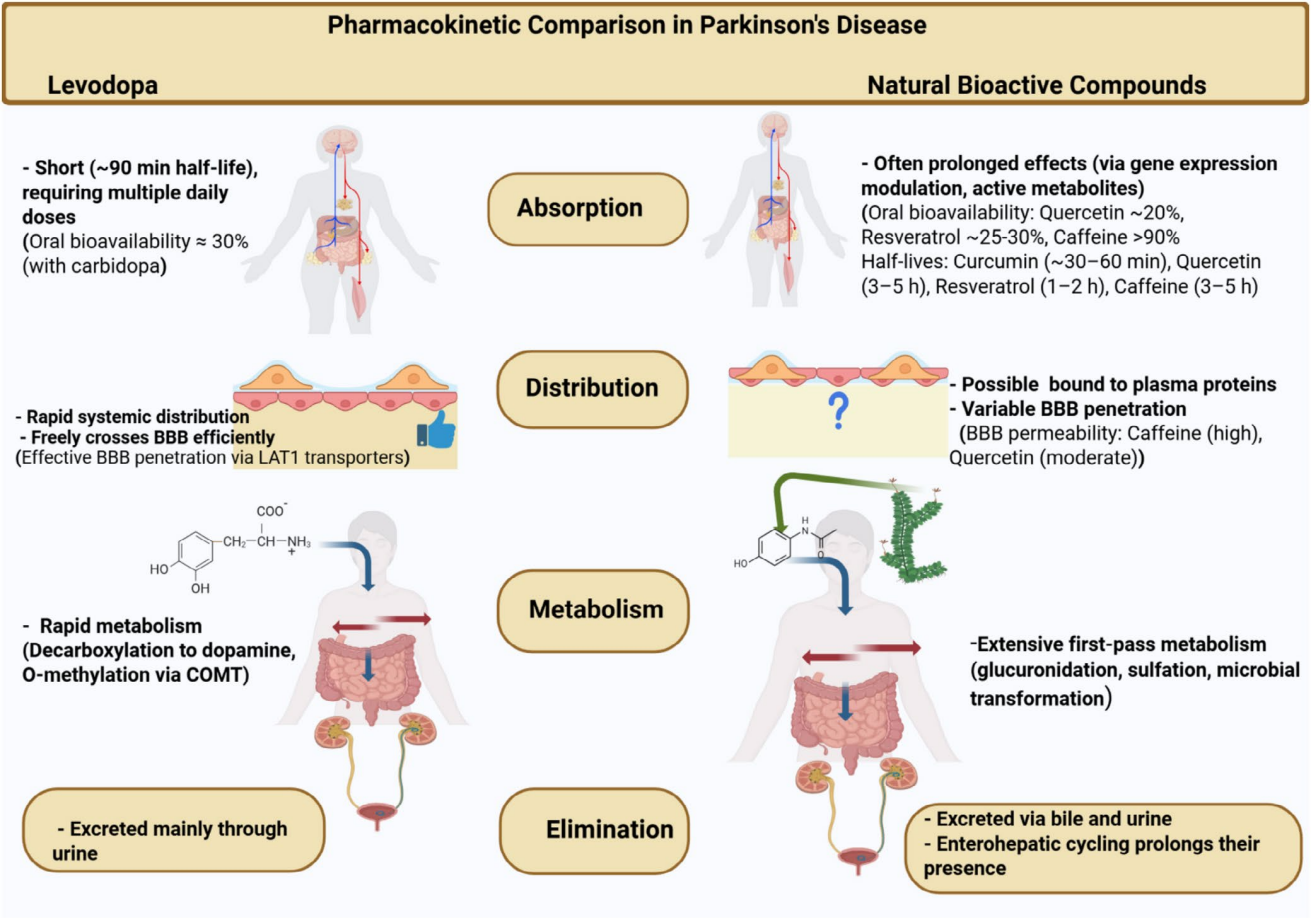
Graphical overview illustrating pharmacokinetic differences between levodopa and selected plant‐derived bioactive compounds used in Parkinson's disease therapy. Specific values are provided for key parameters such as oral bioavailability (e.g., levodopa ≈30%, curcumin < 1%, quercetin ~20%), plasma half‐life (e.g., levodopa ~90 min, resveratrol ~1–2 h), and BBB permeability (e.g., caffeine: High; curcumin: Poor).

Levodopa and plant‐derived compounds also differ markedly in their pharmacokinetic profiles—how they are absorbed, distributed, metabolized, and eliminated (Figure 7). These differences influence their clinical use and effectiveness.

### 9.1 Levodopa Pharmacokinetics

Levodopa is a small amino acid‐like molecule, absorbed primarily in the proximal small intestine via large neutral amino acid transporters [94]. It has rapid absorption but also a short plasma half‐life (~90 min) when given with a peripheral decarboxylase inhibitor [95]. Without enzyme inhibitors, most levodopa would be converted to dopamine in the gut and plasma, never reaching the brain; hence, carbidopa or benserazide is always co‐administered to ensure adequate central bioavailability [96]. Even so, levodopa's concentration in blood rises and falls quickly [97, 98]. In early PD, surviving neurons can store some dopamine, buffering these fluctuations, but as the disease progresses and striatal storage capacity is lost, the short half‐life leads to oscillating brain dopamine levels and the aforementioned wearing‐off and motor fluctuations [96, 97, 98]. Levodopa's pharmacokinetics are also affected by diet [99]. High‐protein meals compete for its transport and can delay or reduce absorption [99, 100]. It does cross the blood–brain barrier efficiently (via LAT amino acid transporters) and is converted to dopamine in remaining dopaminergic terminals and other cells. Metabolism of levodopa is mainly by decarboxylation (to dopamine) and O‐methylation via COMT (to 3‐O‐methyldopa); these metabolites are eventually excreted in urine [92]. The need for multiple daily doses of levodopa (due to its short duration of action) and its sometimes erratic absorption in PD (e.g., delayed gastric emptying in some patients) are challenges in maintaining steady symptomatic control. Advanced delivery systems like continuous intestinal gel infusion or extended‐release formulations have been developed to mitigate these issues [101].

### 9.2 Natural Compounds Pharmacokinetics

The plant bioactives encompass a wide range of chemical structures, so their pharmacokinetics vary widely [102]. A common theme for many polyphenols and flavonoids, however, is poor oral bioavailability [103]. Many polyphenols have large, bulky structures or are highly lipophilic, and thus are not absorbed efficiently from the gut [104]. They may undergo extensive first‐pass metabolism (phase II conjugation in the intestine and liver) which drastically reduces the free (unmodified) compound reaching systemic circulation. For example, curcumin is notoriously poorly bioavailable: in animal studies, only nanogram‐level concentrations of curcumin appear in plasma after oral dosing, and in humans even doses as high as 8 g orally can result in undetectable or extremely low plasma levels [36]. Within an hour of oral intake, curcumin is largely metabolized to glucuronide and sulfate conjugates, and only trace amounts of parent compound can be measured [36]. Resveratrol has better absorption than curcumin but is still rapidly metabolized; its half‐life in plasma is on the order of hours, and it mostly circulates as metabolites (sulfates, glucuronides) [105]. Quercetin (a flavonoid) is often ingested as glycosides and is converted to various phenolic acids by gut microbiota; its bioavailability is moderate but improved when taken with fatty meals or in liposomal formulations [106]. Many flavonoids rely on the gut microbiome to be deconjugated and transformed into smaller metabolites that can be absorbed—a process that introduces high inter‐individual variability. Terpenoids range from small volatile monoterpenes (which are often rapidly absorbed and cleared) to large triterpenes (which may be poorly absorbed) [51]. For instance, ginkgolide B has low oral bioavailability but does cross the BBB once absorbed; ginsenosides like Rg1 are saponins with sugar moieties that make them bulky, and they are metabolized by intestinal bacteria into more absorbable forms. Berberine has notoriously low oral bioavailability (~1% of an oral dose reaches plasma) due to P‐glycoprotein efflux and first‐pass metabolism, but interestingly, berberine still exerts CNS effects, likely through active metabolites or by modulating the gut–brain axis [107]. Recent research shows berberine can cross the BBB to some extent and that nano‐formulations or co‐administration with absorption enhancers can raise its brain levels [44].

### 9.3 Brain Penetration

Levodopa, by design, has good brain penetration (after peripheral decarboxylase inhibition) – a substantial portion of the administered dose reaches the brain and is converted to dopamine [108]. For natural compounds, penetration of the blood–brain barrier is often a critical question. Polyphenols like resveratrol are relatively small and lipophilic and can enter the brain, though in limited amounts (resveratrol's brain‐to‐plasma ratio is low, but due to its potency it may still have central effects) [105, 109]. Curcumin's brain availability is extremely low in free form, which has prompted development of nanoparticles, liposomes, and other delivery systems to ferry it into the brain [36]. Flavonoid metabolites (like smaller phenolic acids) may actually be the active species that reach the brain. Terpenoids vary: some (like borneol or linalool, which are small terpene molecules) cross the BBB easily; others (like celastrol, a large triterpene) might not cross readily but can influence the CNS indirectly via peripheral immune modulation [110]. Notably, caffeine (a plant alkaloid) is highly bioavailable and distributes throughout the body, including the brain, within minutes; its peak brain levels occur ~20–30 min after ingestion [111]. This is one reason caffeine's effects (stimulation, adenosine blockade) manifest quickly. Nicotine also has very high BBB permeability, reaching the brain in seconds when inhaled (e.g., smoking) [112].

### 9.4 Metabolism and Duration of Action

Many natural compounds have longer biological effects than their plasma half‐lives would suggest, either due to active metabolites or lasting changes in gene expression. For instance, a single dose of a polyphenol can activate Nrf2 or other transcription factors in brain cells, leading to sustained upregulation of antioxidant enzymes for hours or days, even if the compound itself has been cleared from the bloodstream [22, 32]. In contrast, the effects of levodopa cease shortly after its concentration decreases, as it directly replaces a neurotransmitter that needs to be continuously available [60, 88]. This fundamental difference implies that natural compounds might require less frequent dosing to achieve a continuous neuroprotective effect, whereas levodopa's dosing frequency is strictly determined by its short pharmacokinetics [101]. However, the downside is that the low bioavailability of many phytochemicals significantly limits their therapeutic use [68, 106]. Researchers are actively developing formulations to enhance this. For example, combining curcumin with piperine (from black pepper) notably improves curcumin's bioavailability by inhibiting its metabolism [36]. Nanotechnology is being utilized to create brain‐targeted delivery systems for curcumin, resveratrol, quercetin, and other compounds to navigate the pharmacokinetic barriers that diminish the efficacy of these substances in vivo [76, 109]. In contrast, levodopa's pharmacokinetics have been extensively refined over decades, incorporating enzyme inhibitors, controlled‐release pills, intestinal gels, and even pumps for continuous subcutaneous delivery to extend its action [89, 100]. Consequently, levodopa can provide symptomatic relief throughout most of the day with the right dosing regimens, though this may lead to increasing regimen complexity in advanced disease. To compare directly: Absorption—levodopa is quickly absorbed but requires an empty stomach for optimal effect [96]; many natural compounds are absorbed slowly or incompletely and may benefit from specialized delivery agents [110]. Distribution—levodopa predominantly travels unbound in plasma and enters tissues readily, while polyphenols often bind to albumin or are sequestered within specific tissues [106]. Metabolism—levodopa's metabolic pathways are well established (dopa decarboxylase, COMT) [6]; plant compounds frequently undergo phase II metabolism (glucuronidation, sulfation) and microbial transformation [68]. Excretion—levodopa's metabolites are expelled in urine; polyphenol metabolites may be excreted in bile and undergo enterohepatic cycling, prolonging their presence [113]. The duration of action for each dose of levodopa is brief (a few hours), while a single dose of some nutraceuticals may offer subtle but prolonged benefits (for instance, an anti‐inflammatory effect lasting beyond the compound's presence in circulation) (Figure 7) [68, 114]. In summary, levodopa offers favorable brain delivery but has an inconveniently short duration of action, whereas many natural compounds possess inherently longer‐lasting effects but encounter challenges in absorption and brain penetration (Figure 7). The field is actively working to address the bioavailability issue surrounding phytochemicals. Future advancements in formulation could render natural compounds more viable as therapeutics, potentially enabling them to reach the brain more consistently and exert beneficial effects that complement levodopa's pharmacokinetic limitations [85, 108].

## 10 Discussion and Future Directions

Levodopa and plant‐based bioactive compounds represent distinct therapeutic strategies for Parkinson's disease: levodopa effectively alleviates symptoms by directly replenishing dopamine, whereas natural compounds potentially modify disease progression by targeting underlying pathological mechanisms [115, 116, 117]. Rather than replacing levodopa, natural compounds may best serve as complementary therapies, addressing aspects such as dopaminergic neuron preservation, oxidative stress reduction, and inflammation control. A combination approach could extend levodopa's optimal therapeutic period, allowing lower doses and thus decreasing dyskinesia risk [14]. Indeed, synergy between levodopa and natural bioactives already has clinical precedent: istradefylline, modeled after caffeine's natural adenosine A_2_A antagonism, is used to reduce levodopa's “off” periods, while green tea's (+)‐catechin demonstrates COMT inhibition, potentially enhancing levodopa's duration of effect [4, 116, 118]. The greatest promise of plant‐derived compounds lies in their potential as disease‐modifying agents. Natural compounds like curcumin or resveratrol could theoretically slow PD progression by preserving dopamine transporter levels or reducing α‐synuclein accumulation. To validate this, rigorous, long‐term clinical trials should evaluate these compounds as adjunctive or preventive therapies in early‐stage or high‐risk populations, assessing biomarkers and clinical outcomes over extended periods [118, 119]. Standardization remains essential, particularly in herbal preparations such as *Mucuna pruriens* , requiring consistent L‐DOPA content and optimized dosage formulations to ensure reproducible therapeutic outcomes and regulatory approval [47]. Finally, translating promising preclinical findings to human PD therapy demands substantial expansion of clinical trials with robust design, adequate sample sizes, and relevant endpoints such as imaging or biochemical markers [38]. Additionally, while natural bioactive compounds generally exhibit favorable safety profiles, long‐term, high‐dose safety and potential drug interactions—especially in elderly PD patients receiving multiple medications—must be systematically evaluated. Comprehensive safety data are crucial for integrating these natural agents confidently into standard PD management regimens [38, 119, 120, 121]. Although preliminary clinical studies have shown promising results for plant‐derived compounds in Parkinson's disease (PD), robust evidence on long‐term efficacy and disease‐modifying potential remains scarce. Future clinical trials may aim for higher methodological quality by integrating the following elements. *Biomarkers for Disease Progression*: Objective markers such as cerebrospinal fluid (CSF) levels of α‐synuclein, neurofilament light chain (NfL), and dopamine transporter imaging (DAT‐SPECT) should be used to monitor neurodegeneration and treatment response [122, 123, 124]. *Extended Follow‐Up Durations*: As most current studies are limited to relatively short durations (e.g., 8–24 weeks), future trials might benefit from extended follow‐up periods (e.g., ≥ 12 months) in order to more reliably assess sustained therapeutic effects and the potential for disease modification [125, 126]. *Standardization of Plant‐Based Interventions*: Given the variability in dosage, purity, and formulation reported in many phytochemical studies, the use of GMP‐grade, standardized extracts or well‐characterized isolated compounds may enhance reproducibility and improve the interpretability of dose–response relationships [127, 128, 129]. *Multicenter and Randomized Design*: Well‐powered, multicenter randomized controlled trials (RCTs), incorporating rigorous blinding and standardized outcome measures, could be critical in validating preliminary findings and supporting the clinical translation of plant‐derived interventions in Parkinson's disease [130, 131].

## 11 Conclusion

In conclusion, leveraging natural bioactive compounds in PD therapy is a promising avenue that seeks to marry symptom control with neuroprotection. Levodopa will likely remain the cornerstone for symptomatic treatment—its immediate and robust effect on motor function is unparalleled. However, the future of PD management may be a multi‐pronged therapy: using levodopa (or dopamine agonists) to manage symptoms, plus plant‐derived compounds to provide continuous neuroprotective support, improve antioxidant defenses, and combat inflammation. This combined approach could extend the period during which patients have a good quality of life and slow the progression of disability. Should any of these compounds show strong disease‐modifying effects in clinical trials, they could even be started in at‐risk individuals to prevent PD (an exciting prospect in preventive neurology). From a research standpoint, the path forward involves intensive preclinical studies to pinpoint mechanisms (e.g., which pathways to target for neuroprotection), innovative drug delivery solutions to improve bioavailability, and well‐structured clinical trials following CONSORT guidelines to provide high‐quality evidence. The interdisciplinary nature of this effort—combining neurology, pharmacology, natural product chemistry, and even nutritional science—is reminiscent of the development of levodopa itself (which was originally derived from *Vicia faba* beans). Just as levodopa's discovery transformed PD care in the 20th century, perhaps plant‐derived therapies will shape the future paradigm of treating Parkinson's disease, aiming not only to treat symptoms but also to change the disease course. Ultimately, it is not a competition between levodopa and natural compounds, but a synergy: levodopa for what it does best—replacing dopamine—and natural bioactives for what they do best—protecting and healing neurons. This complementary strategy holds the best promise for holistic management of Parkinson's disease in the years ahead.

## Author Contributions

E.A. conducted the research, designed and edited the figures, and wrote the first draft of the manuscript. H.A.H. and N.Ö.Ö. supervised the study. All authors reviewed and commented on previous versions of the manuscript. All authors read and approved the final manuscript.

## Ethics Statement

The authors have nothing to report.

## Consent

The authors have nothing to report.

## Conflicts of Interest

The authors declare no conflicts of interest.

## Data Availability

The authors have nothing to report.
